# Understaffing and overprescribing: a register study on the role of locum physicians in antibiotic prescribing patterns in primary care in Northern Sweden

**DOI:** 10.1080/02813432.2025.2571928

**Published:** 2025-10-21

**Authors:** Björn Dagberg, Grzegorz Szparecki, Thorbjörn Lundberg

**Affiliations:** a Vindelns health care centre, Umeå, Sweden; b Backens health care centre, Umeå, Sweden; c Anderstorps health care centre, Skellefteå, Sweden; d Department of Public Health and Clinical Medicine, Umeå University, Umeå, Sweden

**Keywords:** Respiratory tract infections, antibiotics, prescriptions, health workforce, locum physicians

## Abstract

**Introduction:**

Understaffing and dependence on temporary physicians known as locums are common in primary healthcare in Sweden. In this study, we investigate the impact of locum staffing on the quality of antibiotic prescribing for respiratory tract infections. We used two neighbouring cities in northern Sweden with large differences in staffing conditions: Skellefteå, with a 10-fold higher reliance on locum physicians compared to Umeå, which has low locum staffing.

**Methods:**

We used data extracted from patient records for all visits, from the four largest healthcare centres in each of the cities Umeå and Skellefteå, for respiratory tract infections, including the ICD-10 diagnosis code and whether antibiotic treatment was prescribed or not. Data on the contracted hours of locum staff for each healthcare centre were also obtained. Statistical analyses were performed with Spearman correlation, Chi-square and Poisson tests. Logistic regression was used to investigate whether prescribing patterns were affected by the level of relay physicians.

**Results:**

We analysed antibiotic prescriptions for respiratory tract infections based on data from 11,228 patient visits. Regardless of the statistical method used, we found a statistically significant difference, with higher rates of antibiotic prescribing for diagnoses where antibiotics generally are not recommended or where they should be prescribed restrictively. For pneumonia, where antibiotics should normally be prescribed, no difference was seen. The odds of antibiotic prescription for acute upper respiratory tract infection in Skellefteå were 2.5 times higher than in Umeå, with a positive correlation between locum staffing and antibiotic prescriptions for diagnoses where antibiotics should be used restrictively.

**Conclusions:**

Our findings show that a high level of locum staffing and thus insufficient staffing with permanent General practitioners leads to higher rates of inappropriate antibiotic prescribing. This is shown both at the level of individual primary care healthcare centres and at the community level.

## Introduction

Approximately 50% of all antibiotic prescriptions occur in primary care, with respiratory infections being one of the two major reasons for prescribing, the other being urinary tract infections [1]. In Sweden, antibiotic prescriptions declined by 52% between 1992 and 2024, thanks to the national strategic program against antibiotic resistance known as STRAMA [2–4]. However, significant differences in prescribing patterns exist among different physician categories [5]. For instance, locums tend to prescribe a lower percentage of penicillin V compared to more broad-spectrum antibiotics, especially when compared to staff physicians. Additionally, locums prescribe antibiotics more frequently for cough and acute bronchitis in patients below 50 years of age.

Understaffing is a prevalent issue in primary healthcare in Västerbotten County and across Sweden. A recent report indicates that approximately 40% of the need for primary care physicians in Sweden remains unmet [6]. To achieve adequate staffing at the national level, the number of primary care physicians would need to increase from 4,900 to 8,300 [6,7]. Locum physicians (rental physicians) play a significant role in fulfilling healthcare responsibilities, particularly as there are regional differences in adequate staffing. While affluent urban areas and cities hosting medical schools have better staffing situations, remote areas often rely entirely on locums.

The dependence on locums for daily clinical practice introduces challenges. It increases costs (for hiring) and typically only raises physician staffing to the minimal required level. Consequently, permanent general practitioners and residents shoulder an increased workload in comparison to fully staffed primary health care centres (PHCCs). This situation poses risks, including hasty clinical assessments and antibiotic prescriptions ‘just in case’ for unclear scenarios, all in the interest of handling individual cases promptly [8]. Furthermore, locums are often excluded from structural feedback on prescription patterns provided to staff physicians at regular intervals [9]. The disruption of physician-patient continuity, which significantly impacts primary care quality and patient outcomes, is another consequence of locum staffing [10,11].

Although it is well established that individual locums prescribe antibiotics more frequently [5,12], the impact of high levels of temporary staffing and the corresponding shortage of permanent GPs has not been previously studied at the community level. Therefore, the objective of this study is to use locum staffing levels as a proxy for understaffing and analyse how this understaffing influences the quality of care at both the PHCC and community level, focusing specifically on respiratory tract infections and antibiotic prescribing.

## Methods

### Ethics

The study was approved by the regional ethics committee in Umeå (2018/102-32). One-way encrypted ID-numbers were used to ensure confidentiality for individual patients. The analyses were performed on a large number of individual patient visits, and it is not possible to identify information attached to any individual in the final result. Analysis of prescriptions was performed on the specific clinic, and no collected data can be linked to individual prescribing patterns for each clinic’s physicians.

### Data extraction and study population

Data was obtained from the DIVER database, collecting patient and medical data from all patient medical records in the main medical record system, System Cross, in Västerbotten county. Data extracted included date of birth, sex, visit date, diagnosis, visited clinic and whether an antibiotic was prescribed or not. There was no information on which specific antibiotic was prescribed, nor on the name of the prescribing physician or the physician’s employment form. A data set containing all visits in Västerbotten County to clinics using the System Cross Medical Records System was obtained from the DIVER database, with diagnoses for acute upper and lower respiratory tract infection. Data for all visits for patients aged 0–64 were used. Visits for patients 65 years of age and older were excluded because of more complex comorbidities and the considerations associated with late-life care.

The number of patients listed on each PHCC for each year was collected from the DIVER database.

The number of hours of contracted locums (rental physicians) for each PHCC was obtained from the Human Resources department at Region Västerbotten. For the period 2016–2017, there was a centrally procured price per hour, which means that the cost can be converted to contracted time for these years reproducibly. Therefore, these years were used for the analyses.

Eight public sector PHCCs (the four largest in each of the two major cities in Västerbotten County, Umeå and Skellefteå) were selected for analysis. Private PHCCs were omitted as no data for staffing was available. We have chosen to analyse only large health centres to avoid the impact of habits of single individuals who become more pronounced at small units with few physicians. The PHCCs included represent half the population in Umeå and two-thirds of the population in Skellefteå.

Visits resulting in the following diagnoses according to the 10th revision of the International Classification of Diseases and Related Health Problems (ICD-10), diagnoses H6xx, H7xx, J0xx, J1xx and J2xx, were included in the data set. A total number of 11,228 patient visits (visits or revisits not specified) in the years 2016 and 2017, to a physician with these diagnoses, were used for the analyses. Data was analysed for each PHCC and year separately as locum staffing differed for each year. The outcome of individual visits diagnosed with respiratory tract infection was selected for analysis. Analysis of antibiotic prescriptions related to the largest diagnostic groups includes a total of 9,294 of these visits (Table 1).

**Table 1.** Descriptive data on analysed diagnoses for all analysed patients and separately for each city as a proportion of patients receiving antibiotic treatment.

	All		Umeå		Skellefteå	
Diagnoses	Proportion (%)	N	Proportion (%)	N	Proportion (%)	N
All diagnoses	42	9294	34	5495	51	3799
AURTI	8.6	4777	4.4	3140	13	1637
Tonsillitis	82	1149	79	627	85	522
Pneumonia	75	668	73	317	77	351
Sinusitis	59	702	54	353	65	349
AOM 1–12 years	81	1225	77	689	84	536
AOM except 1–12 years	79	773	79	369	78	404

Number of patients (N). AURTI: Acute upper respiratory tract infections. AOM: Acute otitis media.

**Table 2.** Logistic regression coefficients for locum dependence [workhours/listed patient] for each diagnosis, providing the odds for antibiotic prescription after including the patient’s age and gender in analyses.

	*B*	*SE*	*OR* [95% *CI*]
AURTI	1.13	0.136	3.10*** [2.38–4.05]
Sinusitis	1.04	0.259	2.82*** [1.70–4.68]
Tonsilitis	0.799	0.271	2.22** [1.31–3.78]
AOM (1–12 years old)	0.902	0.271	2.47** [1.45–4.19]
AOM (other ages)	0.129	0.279	1.14 [0.658–1.97]

*p < 0.05; **p < 0.01; ***p < 0.001.

B – regression coefficient; SE – standard error of regression coefficient; OR – odds ratio; 95% CI – 95% confidence interval [upper and lower level]; AURTI - Acute upper respiratory tract infections; AOM - Acute otitis media; *p* - significance level.

### Study design

A retrospective register-based study based on data for all visits to a physician at the selected PHCC, for which a diagnosis of infection in the upper and lower airway was coded acute respiratory tract infections (ARTI), between 2016 and 2017. The proportion of antibiotics prescribed was compared between PHCC with low and high locum staffing. Correlation and regression analyses between the level of locum staffing and antibiotic prescription were performed for all diagnoses and subgroups of diagnoses. The subgroups were defined as follows, with their corresponding ICD-10 codes: 1) Acute upper respiratory infection (AURTI): J06.9; 2) Tonsillitis: J03.0, J03.8, and J03.9; 3) Pneumonia: J15.7, J15.9, J18.0, and J18.9; 4) Sinusitis: J01.9; and 5) Acute and non-defined otitis media (AOM): H659, H660, and H669. Analyses of AOM were further refined by separately analysing the age group 1–12 years, due to distinct national guidelines for treatment within this age group. The analysis included ICD-10 codes that were most frequently assigned to patients within each major diagnostic category, ensuring that the analysis focused on the primary diagnoses relevant to the research objectives (Table 1).

### Statistical analysis

All analyses and descriptive statistics were performed using IBM SPSS version 25, R version 4.3.3 and Excel. The proportion of prescribed antibiotics for all diagnoses and for diagnosis groups at each PHCC was calculated. *p* values ≤0.05 were considered statistically significant.

Spearman correlation analysis was performed to assess if there was a relation between the percentage of visits that involved prescription of antibiotics and the number of work hours contracted in each health centre per listed patient. Thus, each data point represented a health centre at any given year, as both variables varied between 2016 and 2017. Non-parametric correlation was chosen because of a limited number of data points and the skewed distribution, especially in the percentage of prescriptions of antibiotics.

Further analysis involved comparison of the four largest health centres in Umeå and Skellefteå in terms of prescription of antibiotics for each selected diagnosis per number of listed patients/year, per number of visits and per locum physician involvement in any given health centre. For these calculations, the Poisson test, chi-squared test and logistic regression were employed, respectively.

We have calculated the number of visits that ended with the prescription of antibiotics for each selected diagnosis, per number of listed patients per year. Because this variable counts the number of events in a given amount of time, the Poisson test is applicable to compare it in two settings (Umeå and Skellefteå). For each value, 95% confidence intervals were calculated.

Chi-squared test was used on data arranged in a 2 × 2 table for each selected diagnosis. We have calculated the number of visits that ended with a prescription of antibiotics (as a positive outcome) and those that did not result in a prescription of antibiotics (negative outcome). We have treated patients in Skellefteå as the exposed group and patients in Umeå as the non-exposed group. Based on these labels, the odds ratio (OR) with 95% confidence intervals was calculated.

Finally, logistic regression analyses for each selected diagnosis were performed. Here, the dependent variable was the prescription of antibiotics assessed as a binary value (for each visit, antibiotics were or were not prescribed). Independent variables included patient age and gender, as well as locum physician involvement in the health centre (counted as the number of contracted work hours per listed patient). Based on these, regression models were built. Their validity was checked with Nagelkerke’s R-squared value, percentage of correctly predicted outcomes and chi-squared tests. For valid models, further analysis on independent variables was performed, namely, if these variables contribute to the predictive value of each model – B-coefficients, significance and odds ratio (OR) with 95% confidence intervals were calculated. Finally, models were double-checked for outliers through analysis of Cook’s distances and leverage values for each case.

Tentatively, we tried to include all patients from all health centres in both cities in this analysis, but analysis of Cook’s distances and leverage values showed that visits to smaller health centres disproportionately influenced the regression models. This posed an analytical challenge, as our data only reflects the general workload of locum physicians for each health centre. At smaller health centres, this could mean that the presence of one or two physicians could significantly alter the regression model. Therefore, we chose to include only the four largest health centres in both cities in the analysis.

## Results

Characteristics of staffing and frequency of patient visits, as well as demographic differences, are described in Supplementary Tables 1 and 2. Demographically, Umeå and Skellefteå differ, with a higher proportion of young adults in Umeå and a relatively lower proportion of elderly individuals. However, there was only a slight difference in the proportion of children and adolescents between the ages of 0 and 19 (Supplementary Table 2). The average number of listed patients per PHCC and the degree of locum staffing are also described in Supplementary Table 1. Notably, the difference in locum staffing between the cities was tenfold, and the health centre with the highest degree of locum staffing in Umeå was below the PHCC with the lowest degree of locum staffing in Skellefteå.

Chi-squared analyses showed that the frequency of antibiotic prescriptions in patients with AURTI was significantly higher (χ^2^(2) = 69.66; *p* < 0.001) in Skellefteå (12.9%) compared to Umeå (4.4%). The odds ratio was 2.53 (95% *CI*: 2.02–3.16), thus patients with AURTI in Skellefteå had 2.53 times greater odds for getting antibiotics prescribed than patients in Umeå. For other diagnoses (AOM, pneumonia, sinusitis, tonsillitis), we did not observe any significant difference. See Table 1 for absolute numbers and proportions.

Analyses using the Poisson test showed that prescriptions per 1000 listed patients/year were significantly lower in Umeå for the following diagnoses: AURTI, AOM, pneumonia and sinusitis. For tonsillitis, there were no significant differences (Table 3).

**Table 3.** Poisson test for comparison of antibiotic prescription in the respective city. The number of visits with antibiotic prescription per 1000 listed inhabitants per year [95% confidence interval].

Diagnoses	Skellefteå	Umeå	*p*
AURTI	2.0 [1.6–2.5]	1.52 [1.0–1.5]	0.002
AOM (all ages)	8.0 [7.4–9]	8.7 [6.4–7.8]	0.05
Pneumonia	2.8 [2.4–3.4]	2.44 [1.6–2.4]	0.005
Sinusitis	2.4 [2.0–2.9]	1.9 [1.2–1.9]	0.002
Tonsillitis	4.7 [4.2–5.4]	5.2 [3.7–4.8]	0.2

AURTI - Acute upper respiratory tract infections; AOM - Acute otitis media; *p* - significance level.

Results of Spearman correlation analyses between antibiotic prescriptions for the respective diagnoses and the level of locum staffing at individual PHCCs are shown in Table 4. The analysis showed a statistically significant positive correlation between higher rates of temporary staffing and increased prescription of antibiotics for acute upper respiratory tract infection, tonsillitis, and otitis in children aged 1–12 years. No significant correlation was found for patients with pneumonia and AOM, except in the 1–12 age group with AOM.

**Table 4.** Spearmann’s correlation between the percentage of visits with antibiotic prescriptions per diagnosis and the degree of locum physician staffing at each health Centre (hours/listed patient).

Diagnoses	R	*p*
All patients	0.585	<0.001
AURTI	0.768	0.001
Sinusitis	0.585	0.02
Tonsilitis	0.655	0.006
AOM age 1–12	0.678	0.004
AOM except age 1–12	0.185	0.5
Pneumonia	0.00741	1.0

AURTI - Acute upper respiratory tract infections; AOM - Acute otitis media; *p* - significance level.

Logistic regression models could be built that significantly predicted prescription of antibiotics based on patients’ age, gender and dependence on locums at the health centre for all diagnoses included in the study except for pneumonia (Table 2, Supplementary Table 3). These models showed that independently of age or gender of the patients, odds for antibiotic prescription increased significantly with staffing provided by locum physicians, measured as work hours per listed patient, for the following diagnoses: AURTI, pneumonia, sinusitis, tonsillitis and AOM (in patients 1–12 years old). Apart from these findings, we also observed that in patients in the age group 1–12 years old with otitis, the odds for prescription of antibiotics decreased with age.

## Discussion

### Main results

Our study shows that increased use of locum physicians at PHCCs, a consequence of understaffing, is associated with higher rates of antibiotic prescribing. Two complementary analytical approaches support this finding. First, a comparison of PHCCs in Umeå and Skellefteå, where locum use is ten times higher, showed a significant difference in prescription rates. Second, correlation and regression analyses across all PHCCs demonstrated a direct association between locum staffing levels and increased antibiotic prescriptions, independent of city. The difference in prescribing is most pronounced for AURTI, a diagnosis for which antibiotics are generally not indicated.

Increased prescribing rates were evident in the overall listed population, the proportion of visits ending with an antibiotic prescription, and the odds of receiving an antibiotic prescription based on locum workload. We also found significant correlations between locum staffing and antibiotic prescribing for tonsillitis, sinusitis and AOM in children 1–12 years, conditions in which antibiotic use should be selective. On the other hand, we found no or minimal significant differences in pneumonia and AOM diagnoses in patients outside the 1–12 age group, where antibiotics should normally be prescribed.

### Generalizability

The two cities in our study, Umeå and Skellefteå, are medium-sized and located in a sparsely populated region of Sweden, separated by 140 km with only small communities in between. Due to this distance, there is minimal patient exchange and commuting among physicians between the cities. Although similar in many respects and representative of Sweden, the cities differ in employment composition: Skellefteå has a larger proportion of residents working in industry, whereas Umeå’s population is more involved in public administration and education. This is reflected in a larger proportion of the population with a university degree in Umeå, but also a lower level of employment due to the large number of students. Median income was identical between the cities. There was a slightly higher proportion of welfare recipients in Skellefteå (16%) compared to Umeå (12%). Overall, socio-economic status is similar between the cities. However, the cities are opposites in terms of primary care physician staffing, with Skellefteå far more dependent on locum physicians to meet healthcare needs. Similar patterns are observed elsewhere in Sweden, with peripheral and rural areas experiencing greater staffing strain, while university towns such as Umeå and affluent urban regions generally have better staffing conditions. Therefore, we believe our results are likely generalizable to primary care settings across Sweden

The age structure also differs between the cities, with Umeå having a larger population of young adults, due in part to its university, where students make up about 30% of the population, of which 60% are women. This results in a higher proportion of young adults, especially young women, in Umeå. To assess whether these demographic differences could affect our results, we applied logistic regression to examine the influence of locum workload independently of age or gender. This analysis showed that demographic factors had a very limited effect on the findings, suggesting that the differences in population composition are unlikely to have impacted our results.

### Comparison with previous studies

Previous research has demonstrated that the propensity to prescribe antibiotics for acute ARTI varies among physicians in different forms of employment. Physicians at private PHCCs exhibit more liberal antibiotic prescribing than physicians at public PHCCs [13]. Locums have higher antibiotic prescribing rates compared to permanently employed physicians [5,12,14], which may be influenced by individual factors (such as access to in-service training) and clinic-specific conditions (such as workload and familiarity with local routines). Research suggests that physicians with stress-related illnesses are more likely to prescribe antibiotics for ARTI [8]. A systematic meta-study from the United States highlights the risk of negative effects on the quality of care associated with stress-related illness and burnout, although this area remains poorly researched [15]. Locums generally face a high workload with frequently booked patients, and more often patients with acute complaints such as infections, leading to a more challenging work environment. Additionally, the workload of the clinic’s regular physicians is likely more demanding due to understaffing, which necessitates the use of locums. Consequently, both permanent employees and locum physicians experience an increased workload in understaffed PHCCs. The societal-level effects observed in this study reflect an aggregate impact, and we cannot distinguish the influence of higher workloads on permanent physicians from those due to locum staffing. Nevertheless, the results clearly show that a higher proportion of locums correlates with inappropriate antibiotic prescriptions, which is in line with the results on the individual level [5,12,14].

### Strengths

The impact of employment type on individual physicians’ antibiotic prescriptions has previously been demonstrated [5,12,14,16,17]. In this study, we have focused on how shortcomings in permanent physician staffing correlate with a lack of stringency in antibiotic prescribing for respiratory tract infections at the local societal level, in terms of a major part of the population in both cities. We aimed to adopt a broader perspective, considering the entire population rather than focusing solely on individual visits and individual physicians. To our knowledge, this has not been studied before. By using the two largest cities in Västerbotten County, with similar sizes and PHCCs of comparable characteristics but a significant difference in permanent physician staffing, we could address how understaffing and dependence on locums affect the quality of care, based on indications of inappropriate antibiotic prescribing.

### Limitations

One important limitation of this study is the lack of information on whether each specific visit was performed by a temporarily employed physician. Therefore, we used proxy variables to address this gap, ultimately selecting the number of locum work hours per listed patient at each health centre and comparing two cities–Umeå and Skellefteå– which differ significantly in their need for locum physicians. Locum physician staffing in Skellefteå accounted for a major proportion of total physician hours, whereas in Umeå it was modest. Across PHCCs in Västerbotten County, the presence of locum physicians unequivocally indicates understaffing. A PHCC could not contract a locum physician unless the shortage corresponded to the absence of more than one full-time permanent physician. We acknowledge that these two cities differ in other respects which we could not control for in this study. One such factor is the age and gender structure (see above), which we could at least partially quantify in regression analyses.

The difference in age distribution between the cities means a slightly higher burden of care in Skellefteå due to its larger elderly population, which may increase the workload for PHCC physicians and potentially influence the results in ways that our primary data limitations prevent us from fully adjusting for.

Another limitation is the absence of underlying diagnostic information in our data, preventing us from drawing conclusions about the diagnostic accuracy for each condition or assessing differences in diagnostic precision between PHCCs. Additionally, we could not control for whether there are differences in return visit frequency to follow up on recovery. However, this likely plays a greater role for conditions such as pneumonia, and the non-significant differences observed in antibiotic prescriptions for pneumonia may relate to differences in the number of return visits for follow-up. It is also likely that the frequency of revisits for milder respiratory infections was lower.

## Clinical implications and future research

The reliance on locum physicians and understaffing has been shown to significantly impact the quality of care, particularly in the context of antibiotic prescribing for ARTIs. This over-prescription not only contributes to the growing problem of antibiotic resistance but also indicates a deviation from serving the population with qualitative care. Healthcare policymakers and administrators should prioritise strategies to reduce dependency on locum staffing and understaffing. This could include offering competitive salaries, providing professional development opportunities, and creating a supportive work environment to attract and retain permanent GPs. Ensuring consistent, equal and high-quality care across primary care is essential to mitigate the risks associated with inappropriate antibiotic use and to promote better health outcomes at the community level.

Future research should focus on exploring interventions that can effectively reduce the reliance on locum physicians in PHCCs. Studies could investigate the impact of various staffing models on healthcare quality and patient outcomes. Additionally, research should aim to identify specific factors that contribute to the higher rates of antibiotic prescribing among locum physicians and develop targeted strategies to address these issues. Investigating supporting actions to keep locum staff updated on current guidelines and treatment recommendations is crucial. This could include regular training sessions and access to updated clinical digital resources in various forms. Furthermore, exploring the role of artificial intelligence in providing real-time support and decision-making assistance to locum physicians could be beneficial. AI-driven tools could help ensure adherence to treatment guidelines and improve the overall quality of care.

Longitudinal studies examining the long-term effects of staffing changes on antibiotic resistance patterns and overall healthcare quality would provide valuable insights. Furthermore, comparative studies between regions with varying levels of locum staffing could help elucidate the broader implications of staffing disparities on public health.

By addressing these research gaps, we can develop evidence-based policies and practices that ensure equitable and high-quality healthcare for all communities, regardless of staffing challenges.

## Conclusion

Our study demonstrates a clear link between understaffing and reliance on locum staffing, and the over-prescription of antibiotics for AURTI, AOM in children aged 1–12 years, tonsillitis, and sinusitis. These differences are strongly associated with the degree of locum staffing. Maintaining fully staffed PHCCs with permanent GPs is crucial to prevent iatrogenic harm and ensure equitable healthcare quality.

## Supplementary Material

Supplemental Material

## References

[1] European Centre for Disease, P., et al. Antimicrobial consumption and resistance in bacteria from humans and food-producing animals: fourth joint inter-agency report on integrated analysis of antimicrobial agent consumption and occurrence of antimicrobial resistance in bacteria from humans and food-producing animals in the EU/EEA JIACRA IV - 2019-2021. Efsa J. 2024;22(2):e8589. doi: 10.2903/j.efsa.2024.8589. 38405113 PMC10885775

[2] Swedres-Swarm. Consumption of antibiotics and occurence of resistance in Sweden. Solna, Sweden: Public Health Agency of Sweden and National Veterinary Institute; 2024.

[3] Mölstad S, Löfmark S, Carlin K, et al. Lessons learnt during 20 years of the Swedish strategic programme against antibiotic resistance. Bull World Health Organ. 2017;95(11):764–773. doi: 10.2471/BLT.16.184374. 29147057 PMC5677604

[4] Tyrstrup M, Beckman A, Mölstad S, et al. Reduction in antibiotic prescribing for respiratory tract infections in Swedish primary care- a retrospective study of electronic patient records. BMC Infect Dis. 2016;16(1):709. doi: 10.1186/s12879-016-2018-9. 27887585 PMC5124268

[5] Tell D, Engström S, Mölstad S. Adherence to guidelines on antibiotic treatment for respiratory tract infections in various categories of physicians: a retrospective cross-sectional study of data from electronic patient records. BMJ Open. 2015;5(7):e008096. doi: 10.1136/bmjopen-2015-008096. PMC4513445 26179648

[6] The National Board of Social Affairs and Health. Personalstatistik innom primärvården. Delrapport. 2023-3-8459:17-18.

[7] Swedish Medical association. *Läkarförbundet granskar: God och nära vård*. 2024; 10. Available from: https://slf.se/app/uploads/2024/12/lakarforbundet-granskar-gonv-24-webb-1.pdf.

[8] Brady KJS, Barlam TF, Trockel MT, et al. Clinician distress and inappropriate antibiotic prescribing for acute respiratory tract infections: a retrospective cohort study. Jt Comm J Qual Patient Saf. 2022;48(5):287–297. doi: 10.1016/j.jcjq.2022.01.011. 35489803

[9] Hawes L, Buising K, Mazza D. Antimicrobial stewardship in general practice: a scoping review of the component parts. Antibiotics (Basel). 2020;9(8):1–13. doi: 10.3390/antibiotics9080498. PMC7459857 32784918

[10] Sandvik H, Hetlevik Ø, Blinkenberg J, et al. Continuity in general practice as predictor of mortality, acute hospitalisation, and use of out-of-hours care: a registry-based observational study in Norway. Br J Gen Pract. 2022;72(715):e84–e90. doi: 10.3399/BJGP.2021.0340. 34607797 PMC8510690

[11] Skarshaug LJ, Kaspersen SL, Bjørngaard JH, et al. How does general practitioner discontinuity affect healthcare utilisation? An observational cohort study of 2.4 million Norwegians 2007-2017. BMJ Open. 2021;11(2):e042391. doi: 10.1136/bmjopen-2020-042391. PMC7888374 33593777

[12] Borek AJ, Pouwels KB, van Hecke O, et al. Role of locum GPs in antibiotic prescribing and stewardship: a mixed-methods study. Br J Gen Pract. 2022;72(715):e118–e127. doi: 10.3399/BJGP.2021.0354. 34990397 PMC8763197

[13] Llor C, Bjerrum L. Antimicrobial resistance: risk associated with antibiotic overuse and initiatives to reduce the problem. Ther Adv Drug Saf. 2014;5(6):229–241. doi: 10.1177/2042098614554919. 25436105 PMC4232501

[14] Grigoroglou C, Walshe K, Kontopantelis E, et al. Comparing the clinical practice and prescribing safety of locum and permanent doctors: observational study of primary care consultations in England. BMC Med. 2024;22(1):126. doi: 10.1186/s12916-024-03332-z. 38532468 PMC10967114

[15] Tawfik DS, Scheid A, Profit J, et al. Evidence relating health care provider burnout and quality of care: a systematic review and meta-analysis. Ann Intern Med. 2019;171(8):555–567. doi: 10.7326/M19-1152. 31590181 PMC7138707

[16] Ferguson J, Walshe K. The quality and safety of locum doctors: a narrative review. J R Soc Med. 2019;112(11):462–471. doi: 10.1177/0141076819877539. 31710823 PMC6851536

[17] Ferguson J, Stringer G, Walshe K, et al. Locum doctor working and quality and safety: a qualitative study in English primary and secondary care. BMJ Qual Saf. 2024;33(6):354–362. doi: 10.1136/bmjqs-2023-016699. PMC11103325 38627099

