# Supplementary material for: Understaffing and overprescribing: a register study on the role of locum physicians in antibiotic prescribing patterns in primary care in Northern Sweden
Source: Scand J Prim Health Care. 2025 Oct 21;44(1):1–9. doi: 10.1080/02813432.2025.2571928 (PMC12918301; doi:10.1080/02813432.2025.2571928)
Supplement: Supplemental Material [file IPRI_A_2571928_SM6434.docx]

**Supplement table 1**

Descriptive data for the healthcare centres in each city.

**Supplement table 2**

Descriptive data for the age distribution in each city.

|  | | 2 016 | | | | 2 017 | | | |
| --- | --- | --- | --- | --- | --- | --- | --- | --- | --- |
|  |  | | | | |  | | | |
|  | | Umeå | | Skellefteå | | Umeå | | Skellefteå |  |
| Listed patients per age group | | N | (%) | N | (%) | N | (%) | N | (%) |
| All | | 59 280 | 100 | 47 585 | 100 | 60 246 | 100 | 47 874 | 100 |
| 0-6 | | 5 548 | 9 | 3 585 | 8 | 5 543 | 9 | 3 617 | 8 |
| 7-19 | | 8 213 | 14 | 6 630 | 14 | 8 618 | 14 | 6 695 | 14 |
| 20-49 | | 28 371 | 48 | 17 842 | 37 | 28 738 | 48 | 17 853 | 37 |
| 50-64 | | 8 439 | 14 | 9 087 | 19 | 8 528 | 14 | 9 156 | 19 |
| 65-79 | | 6 437 | 11 | 7 561 | 16 | 6 515 | 11 | 7 663 | 16 |
| 80+ | | 2 272 | 4 | 2 880 | 6 | 2 304 | 4 | 2 889 | 6 |
|  | |  |  |  |  |  |  |  |  |

**Supplementary table 3**

Logistic regression models for respective diagnosis providing the odds for antibiotic prescription based on locum dependence, patients age and gender.

|  |  |  |  | 95% *CI* | |  |
| --- | --- | --- | --- | --- | --- | --- |
|  | *B* | *SE* | *OR* | *LL* | *UL* |  |
| AURTI (χ2(3) = 48,96; p < 0,001) | | | | | |  |
| Locum dependence [workhours/listed patient] | | 1.1 | 0.13 | 3.1*** | 2.4 | 4.1 |
| Gender (F vs. M) | | 0.11 | 0.12 | 1.1 | 0.89 | 1.4 |
| Age [years] | | 0.01 | <0.01 | 1.0 | 1,0 | 1.0 |
| Constant | | -3.1 | 0.13 |  |  |  |
| Sinusitis (χ2(3) = 20.52; p < 0.001) | | | | | | |
| Locum dependence [workhours/listed patient] | | 1.0 | 0.26 | 2.8*** | 1.7 | 4.7 |
| Gender (F vs. M) | | 0.096 | 0.17 | 1.1 | 0.79 | 1.5 |
| Age [years] | | 0.010 | 0.01 | 1.0 | 1.0 | 1.0 |
| Constant | | -0.44 | 0.27 |  |  |  |
| Tonsilitis (χ2(3) = 11.55; p = 0.009) | | | | | | |
| Locum dependence [workhours/listed patient] | | 0.80 | 0.27 | 2.2** | 1.3 | 3.8 |
| Gender (F vs. M) | | -0.18 | 0.15 | 0.83 | 0.61 | 1.1 |
| Age [years] | | 0.01 | 0.01 | 1.0 | 1,0 | 1.0 |
| Constant | | 1.3 | 0.19 |  |  |  |
| AOM (1-12 years old) (χ2(3) = 20.75; p < 0.001) | | | | | | |
| Locum dependence [workhours/listed patient] | | 0.90 | 0.27 | 2.5** | 1.4 | 4.2 |
| Gender (F vs. M) | | -0.10 | 0.15 | 0.90 | 0.67 | 1.21 |
| Age [years] | | -0.080 | 0.026 | 0.92** | 0.88 | 0.97 |
| Constant | | 1.6 | 0.18 |  |  |  |
| AOM (other ages) (χ2(3) = 28.93; p < 0.001) | | | | | | |
| Locum dependence [workhours/listed patient] | | 0.13 | 0.28 | 1.1 | 0.66 | 2.0 |
| Gender (F vs. M) | | 0.01 | 0.17 | 1.0 | 0.71 | 1.4 |
| Age [years] | | -0.029 | 0.01 | 0.97*** | 0.96 | 0.98 |
| Constant | | 2.2 | 0.25 |  |  |  |

_*_*_p_*_< 0.05; **_*_p_* _< 0.01; ***_*_p_* _< 0.001_
